# The relationship in early childhood body composition and physical activity levels regarding fundamental motor skill development

**DOI:** 10.1186/s12887-023-04298-2

**Published:** 2023-09-13

**Authors:** Katherine E. Spring, Alexandra V. Carroll, Danielle D. Wadsworth

**Affiliations:** 1 School of Kinesiology, Auburn University, Auburn, AL USA; 2 Division of Population and Public Health Science, Pennington Biomedical Research Center, Baton Rouge, LA USA

**Keywords:** Fat-Free Mass, Fundamental Motor Skills, Preschool-aged

## Abstract

**Background:**

Fundamental motor skills (FMS) are considered essential for sport participation and might be deficit in obese children. While evidence indicates that physical activity (PA) levels impact motor skill development, the relationship between body composition, PA, and motor competence, particularly in early childhood, is not thoroughly understood. We aimed to determine if PA, fat mass (FM), and fat-free mass (FFM) are predictors of FMS.

**Methods:**

Preschoolers (n = 47) from two preschools were assessed for FMS, PA, and body composition. Peabody Developmental Motor Scales (PDMS-2) subscale scores were used to assess FMS. PA was assessed with a wrist-worn accelerometer for five days during school. FM and FFM were measured with foot-to-foot bioelectrical impedance.

**Results:**

Linear regressions indicate significant models for stationary skills (SS) (F = 4.57, *p* = .004) and object manipulation skills (OMS) (F = 4.66, *p* = .003). FFM was the only significant predictor of SS (*t* = 3.98, *p* < .001) and OMS (*t* = 3.50, *p* = .001). FM and all intensities of PA were nonsignificant predictors in all models.

**Conclusions:**

These results indicate that interventions that target improving or maintaining FFM may improve FMS.

## Background

Fundamental motor skills (FMS) are the building blocks of movement necessary to participate in complex sports actions [1–3]. FMS are developed in early childhood and include locomotor skills (LS)(hopping, running, skipping, leaping), object manipulation skills (OMS)(catching, kicking), and stability (balancing) [1]. Children’s FMS competency is a growing concern in several countries as greater FMS competency is associated with higher amounts of physical activity (PA) in children and adolescents [1]. Furthermore, mastery of FMS contributes to a child’s physical, cognitive and social development [4, 5]. Initial research also indicates that FMS competency might be deficit in obese children [6].

Approximately 19.3% of US children and adolescents aged 2–19 are considered obese [7]. Furthermore, children in early childhood aged 2–5 years have seen the greatest increases in obesity rates over the last ten years [7]. COVID-19 lockdowns may present a greater need for public health interventions to address unhealthy body mass index (BMI) in children [8]. Childhood obesity is defined as BMI at or above the age-and-gender-specific 95th percentile [9]. Unlike adults, there is no internationally accepted cut-off for excess body fat (BF) percentage in children. Despite this, some studies have suggested that above 30% BF for girls and 25% BF for boys are associated with less favorable health outcomes [10–12]. Obesity is associated with an increased risk of several chronic diseases, including cardiovascular disease [13, 14] and insulin resistance [9]. Moreover, the drastic increases in early childhood obesity rates are notably disquieting as research has found that obesity established in early childhood continues across several decades [15], and obese children are more at risk for adult obesity [16]. One established pathway to reducing the risks associated with obesity is PA.

PA is a complex and multidimensional behavior that involves human movement, resulting in physiological attributes, including increased energy expenditure and improved physical fitness [17]. Previous literature suggests that participation in PA, specifically moderate-to-vigorous PA, for the minimum recommendations reduces the risk of several chronic diseases [13, 14]. However, the current PA guidelines for early childhood advise that children should be encouraged to move and engage in diverse activities at various intensities [18]. As PA is a complex and multidimensional behavior, researchers have also found that PA participation can have a cascading effect on multiple areas of development. In early childhood, PA is associated with improved obesity status [19] and motor skill development [5, 20, 21].

The relationship between body composition, PA, and motor competence is not thoroughly understood, particularly in early childhood. Research currently indicates that FMS competency is predictive of lifetime physical activity levels [22, 23]. Thus stressing the importance of understanding the relationship between body composition, PA, and motor competence in early childhood. To date, researchers have postulated that PA levels were the main driver of motor skill development in early childhood [23]. However, a recent systematic review of longitudinal evidence found inconclusive evidence for a pathway from motor competence to PA and no evidence to suggest that PA predicts motor competence [24]. Further evidence suggests a strong negative bidirectional relationship between motor competence and weight status [24]. Unfortunately, most studies rely on BMI, limiting the understanding of the relationship between body composition and FMS in preschoolers. While BMI is an often accepted tool used as a stand-in measure for assessing weight status [25], it does not account for body composition or examine body fat distribution [26]. This is troubling as research indicates that fat distribution and body composition are considered risk factors for children’s health [27]. Therefore, a better understanding of the driving relationships between body composition, PA, and motor competence in early childhood, is warranted to determine a pathway for successful intervention strategies.

Few studies have examined the relationship between body composition and FMS. In 2017, Musalek and colleagues aimed to understand if higher amounts of fat mass (FM) in 3-6-year-olds were associated with FMS impairments [28] and found that the amount of body fat (BF) was not a significant predictor of FMS competence. However, Musalek and colleagues failed to examine whether fat-free mass (FFM) could significantly predict FMS competency. One recent study aimed to address this gap in the literature by examining different body composition components (i.e., FM, FFM) associated with different elements of FMS competency. Webster and colleagues [29] found that preschoolers with higher FFM had lower locomotor skill scores and TGMD-2 scores. At the same time, there was no relationship between body composition and object manipulation. However, this study did not include PA, which was previously linked to FMS [29]. As obesity [15], proficiency of FMS [3], and levels of PA [30, 31] established in early childhood continues across several decades, the relationship between these variables should be examined for targeted intervention strategies. Furthermore, this information may explain discrepancies among early childhood FMS and PA interventions. Thus, this study aimed to examine how body composition and PA contribute to FMS. We hypothesized that body composition would significantly predict higher FMS scores. Based on current literature, we hypothesized that greater levels of FFM would predict higher FMS scores; however we were unable to hypothesize a direction for FM. Furthermore, we hypothesized that PA levels, specifically moderate-to-vigorous PA, would significantly predict higher FMS scores.

## Methods

### Participants

**Fig. 1:**
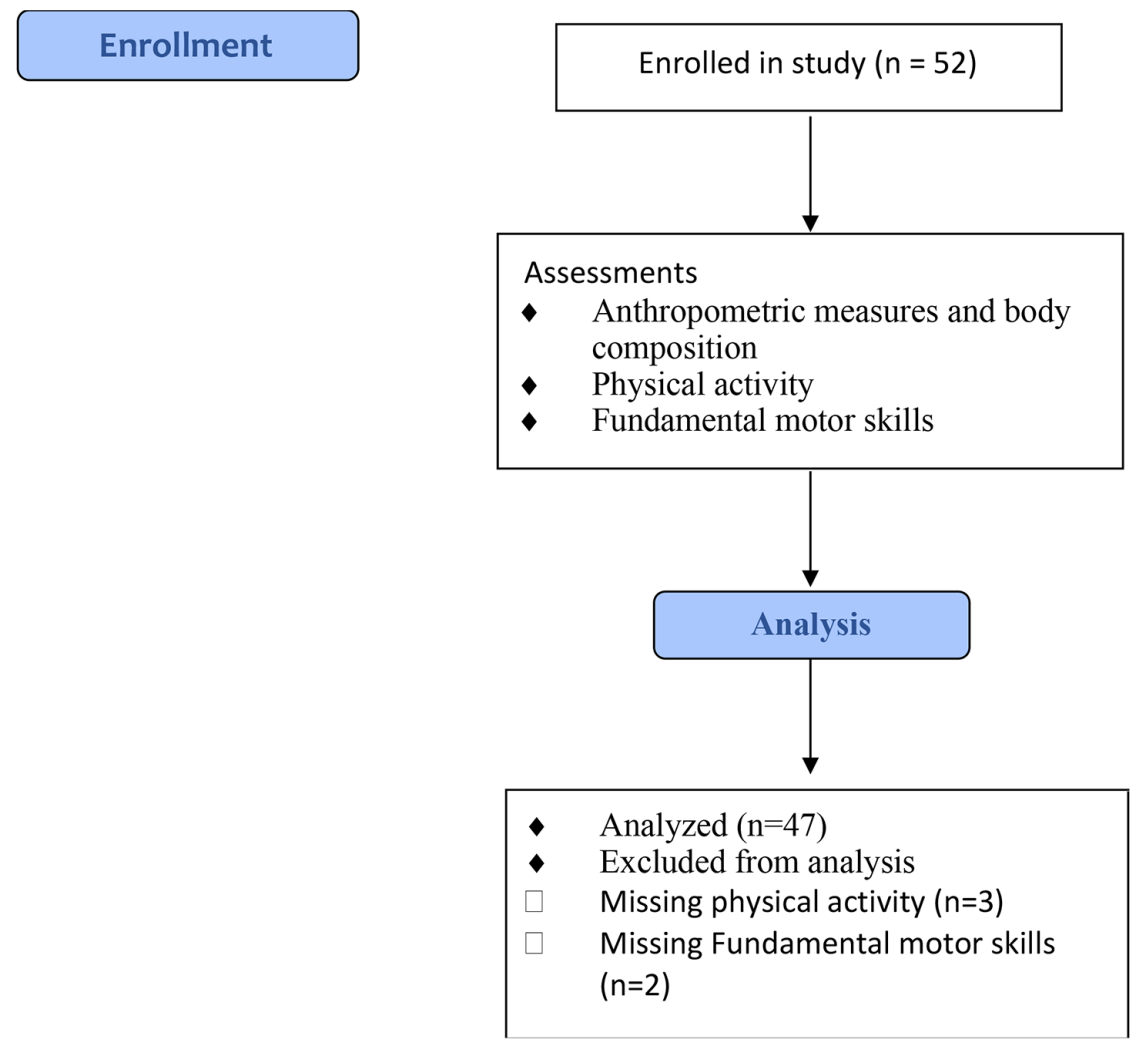
Participant Flow Diagram

Participants were recruited from two local preschool centers. These centers provide all-day childcare services for 6-week-old infants through four-year-old preschoolers. Only children in the 3–4-year-old classes were eligible for participation in this study, as all can locomote independently. This study was approved by the Human Subjects Research Institutional Review Board at Auburn University under protocol number 10–217 MR 1009. Parental informed consent and child assent were obtained prior to data collection. All methods were carried out in accordance with relevant guidelines and regulations. Demographic variables were collected from the parents and preschools. The participants (n = 52) enrolled in the study had a mean age of 3.80 ± 0.714 years. Additionally, the participants were primarily females (51.9%), and most were Caucasian (76.9%). The final analysis included 47 participants with a mean age of 3.81 ± 0.686. Figure 1 displays the participant flow diagram.

### Anthropometric measures and body composition

Height was assessed according to standard procedure [32], utilizing a portable stadiometer. Participants were asked to stand erect, without shoes, with their heads in the Frankfort horizontal plane. All measurements were recorded to the nearest millimeter for height. Body mass was assessed with a digital scale equipped with bioelectrical impedance (BIA)(SC-331 S, Tanita Corporation. Tokyo, Japan). This scale has a recommended minimum age of five years; however, previous work has measured FM, FFM, and fat percentage in preschool-age children using similar Tanita scales (TBF-410GS [33] and SC-331 S [29]). In addition to BIA, BMI and obesity status were determined using age-and-gender-specific growth charts [34].

### Physical activity

In order to assess PA, each participant wore an Actigraph GTX3 (Pensacola, FL) accelerometer on their non-dominant wrist for five school days. Research personnel attached the accelerometer each morning upon participant arrival and removed it before departure. Time on and off was documented for wear time adjustments. Data from the accelerometers was downloaded and analyzed in Actilife 6 (Version 6.13.4). Butte cut points [35] in 10-second epochs were used to categorize activity into light (LPA) or moderate/vigorous physical activity (MVPA). Individual filters based on accelerometer time on and off were used for each participant.

Additionally, researchers examined the attendance records to determine the typical day length for each participant. If the monitor was worn for less than half of a child’s typical attendance time, that day was removed from the final analysis. Participants generally were at school for an average of 492 min (approximately 8 h). If a child wore the monitor for fewer than 246 min (about 4 h), that day was removed from the analysis. The final analysis did not include participants with less than three days of monitoring. The percentage of time spent in LPA and time spent in MVPA were utilized in the final analysis to account for differences in daily wear time (i.e., came to school late, left early).

### Fundamental motor skills

The Peabody Developmental Motor Scales, 2nd Edition (PDMS-2) assessed FMS. The first author administered the PDMS-2 to all participants. Prior to any data collection, the first author was trained by a qualified expert. This training included reviewing the assessment and provided manuals, as well as video review. The PDMS-2 is a norm-and-criterion-referenced fine and gross motor assessment for children aged birth – 5 years. Reliability and validity are reported in the test manual with high coefficients for content sampling (0.89-0.96), time sampling (0.89-0.94), and interrater reliability (0.89-0.96) [36].

This assessment tool consists of six subsections - two fine motor assessments (grasping and visual-motor integration) and four gross motor assessments (reflexes, stationary, locomotor, and object manipulation). Testing for each child is individualized based on developmental age-based milestones for five subtests (grasping, visual-motor integration, stationary, locomotor, and object manipulation). Only the appropriate gross motor assessments for our sample were administered for this study which include: Stationary Skills (SS), Locomotor Skills (LS), and Object Manipulation Skills (OMS). Scores for each task were scored on three levels: performed the task correctly = 2, performed tasks partially = 1, and did not execute the developmental criteria correctly = 0. The sum of points comprises the raw scores for each subscale [36]. Raw scores ranges for each subscale are 0–60 for SS, 0-178 for LS, and 0–48 for OMS. Using age-appropriate tables in the testing manual [36], researchers converted the raw scores to standardized scores (0–20) and summed them to determine the gross motor quartile (GMQ), which had a range of 41–164.

### Data analysis

Statistical software (SPSS, Version 28.0) was used to analyze all data. Descriptive statistics were calculated for all variables. G*power indicated a required sample size of 36 for a small effect (0.35) and 77 for a medium effect to achieve a power of 0.80. Probability values of *p* < .05 were considered significant.

Linear regressions were examined to understand the relationships between FM, FFM, percentage of LPA, percentage of MVPA, and FMS (SS, LS, OMS, GMQ). Four separate models were examined: Model 1 examined body composition, PA, and SS; Model 2 examined body composition, PA, and LM. Model 3 examined body composition, PA, and OMS, and Model 4 examined body composition, PA, and GMQ.

## Results

Table 1 presents demographic variables. Based on BMI, the majority (85.1%) of the participants were classified as healthy weight. Five participants (10.6%) were classified as overweight or obese, and two (4.3%) were underweight. Additionally, 59.6%, 78.6%, 59.6%, and 66.0% of the participants scored in the average category for SS, LS, OMS, and GMQ, respectively.

**Table 1:** Demographic variables

	Mean	Standard Deviation
Weight	15.66 kg	2.08 kg
Height	100.41 cm	5.70 cm
BMI	15.50 kg/m^2^	1.15 kg/m^2^
BMI %	44.51%	28.06%
Body Fat %	20.79%	2.92%
Fat Mass	3.29 kg	0.80 kg
Fat-Free Mass	12.37 kg	1.46 kg
Stationary Skills	48.16 (out of 60)	6.46
Locomotor Skills	145.8 (out of 179)	27.73
Object Manipulation Skills	34.88 (out of 48)	6.46
Gross Motor Quotient	96.32 (out of 164)	12.57

Notes: SS, LS, and OMS, are the mean of raw scores, which are then used to calculate GMQ. There is no unit of measure for these four items.

### Regression results

Model 1 examined raw SS scores with FM, FFM, LPA, and MVPA as predictors. These predictors explained 30.3% of the total variance, F(4,42) = 4.66, *p* = .003. Only FFM contributed significantly (*p* < .001) to SS scores in this model.

Model 2 assessed if FM, FFM, LPA, and MVPA were significant predictors of raw LS scores. After all the predictors were entered, this model was insignificant (F(4,42) = 2.25, *p* = .079).

Model 3 assessed raw OMS scores as the dependent variable, and FM, FFM, LPA, and MVPA were all entered as predictors and explained 30.7% of the total variance, F(4,42) = 4.65, *p* = .003. Upon further examination, it was determined that only FFM contributed significantly (*p* = .001).

Finally, Model 4 examined GMQ with FM, FFM, LPA, and MVPA entered as predictors. No statistical significance was found for this model (F (4,42) = 0.637, *p* = .639). See Table 2 for full regression results.

**Table 2:** Regression results

	F	*df*	r^2^ Δ	*p*	ß	*p*	95% CI
**SS**	4.66	4,42	0.307	0.003			
*FM*					− 0.345	0.058	-2.34 – 0.043
*FFM*					0.701	< 0.001	0.622–1.92
*LPA*					0.072	0.586	− 0.185 – 0.323
*MVPA*					− 0.026	− 0.192	− 0.305 – 0.252
**LS**	2.25	4,42	0.177	0.079			
*FM*					− 0.233	0.234	-10.07–2.53
*FFM*					0.525	0.010	1.18–8.03
*LPA*					0.062	0.666	-1.05–1.63
*MVPA*					− 0.005	0.978	-1.49–1.50
**OMS**	4.65	4,42	0.307	0.003			
*FM*					− 0.097	0.588	-1.68 – 0.252
*FFM*					0.619	0.001	0.526–1.965
*LPA*					− 0.086	0.514	− 0.373 – 0.190
*MVPA*					0.084	0.535	− 0.213 – 0.405
**GMQ**	0.637	4,42	0.057	0.639			
FM					− 0.317	0.133	-4.87 – 0.666
FFM					0.157	0.452	− 0.938–2.07
LPA					− 0.049	0.749	− 0.683 – 0.495
MVPA					0.056	0.724	− 0.532 – 0.760

## Discussion

The study aimed to examine preschool children’s associations between PA, body composition, and FMS. Researchers hypothesized that body composition would significantly predict higher FMS scores. Furthermore, we hypothesized that PA levels, specifically moderate-to-vigorous PA, would significantly predict higher FMS scores. Results indicated that particular aspects of body composition are significantly associated with certain aspects of FMS assessed by PDMS-2 partially supporting our hypotheses. Specifically, we found that greater amounts of FFM were associated with significantly higher SS and OMS. Furthermore, the findings of this study suggest that MVPA and FM may not predict FMS competency for 3-5-year-old children, which does not support our second hypothesis.

The results of the current study contradict those of Webster and colleagues, who found that children with higher amounts of FFM had lower LS scores as well as total TGMD-2 scores [29]. Westerterp and colleagues noted that children with greater FFM often have greater FM [37], which might explain why the FFM was detrimental in the Webster study. However, this does not explain the findings of the current study. It is possible that greater amounts of FFM allowed participants to have better muscle balance when performing the stability assessments. The literature regarding body composition and OMS has mixed results. While Webster in colleagues [29] did not find any associations between body composition and OMS, Matarma [38] and Slotte [39] did find significant differences in body composition and OMS. Matarma found that overweight children performed worse on body coordination, strength, and agility tasks when compared to healthy-weight children [38]. Likewise, Slotte and colleagues found that 8-year-old overweight children had significantly lower OMS than normal-weight children measured by DEXA [39]. It is important to note that different motor assessments were utilized throughout these studies and in different age groups, which may account for variations in the results. Another study from our lab found that a FMS intervention was effective at delaying the addition of FM in a low socioeconomic preschool [40]. While the current study took place in a different preschool setting from our previous study, it does not negate the importance of FMS in obesity prevention or measuring body composition in preschool studies. In fact, current research indicates that early childhood years, specifically children aged 3–5 years, have seen significant increases in obesity and severe obesity from 2013 to 2014 to 2015–2016 [41]. These early years are considered a critical period for growth and development and impact obesity status later in life as overweight and obese children at the age of five are more likely to be obese by fourteen [42]. Additionally, there is evidence that motor competence tracks through childhood into adolescence, and children with higher levels of motor competence are less likely to see declines in physical activity levels [43, 44]. As early childhood is a time of rapid growth and development [45], continued investigations into how excess fat impacts not only FMS and PA, but additional areas of growth and development, are necessary.

Overall, the literature has mixed results regarding body composition and FMS. While some studies have found that greater amounts of FFM are associated with poorer FMS, our results indicated the opposite. Our results may be explained by the relationship between FFM and aerobic fitness [46]. Moreover, FMS competency is positively associated with cardiorespiratory fitness [47], which may be a more consistent indicator of adolescent obesity than PA [48]. However, there is insufficient data to understand the relationship between fitness, body composition, and FMS in young children. Further research should examine these relationships.

One interesting finding of our study was that neither LPA nor MVPA was related to FMS. While research has long considered that in early childhood, PA levels were the main driver of motor skill development [23], recent studies have disputed this association. Barnett and colleagues recently discussed inconclusive evidence for a pathway from motor competence to PA and no evidence to suggest that PA predicts motor competence [24], thereby supporting the findings of our study.

### Limitations

The current study is not without limitations that require consideration when interpreting the results. First, the achieved power for this study was 0.55, which is below the recommended level of power and limits our findings. Second, due to the cross-sectional design, we cannot determine directionality. Third, this study utilized a Tanita scale with a cut-off of five years of age. However, previous work has measured FM, FFM, and fat percentage in preschool-age children using similar Tanita scales (TBF-410GS [33] and SC-331 S [29]. Finally, although this study measured PA at school for over eight hours, we did not measure PA for 24 h. This study was conducted during the COVID-19 pandemic and we were limited in our interaction with the children, families, and what could be taken home from the classrooms. Future studies would benefit from a larger sample and a longitudinal design to better understand the relationship between body composition, PA, and FMS in preschoolers. It would also be beneficial to measure PA for 24 h to understand how PA habits might impact FMS in preschoolers.

## Conclusions

This cross-sectional design study examined the relationships between body composition, PA, and FMS. Results indicated that greater FFM was associated with higher stationary and OMS. Fat mass, LPA, and MVPA were not significant predictors in any model. Future studies would benefit from larger sample sizes. Likewise, it would be prudent to conduct a longitudinal design to understand how these variables change over time in preschoolers and how these changes impact the relationships.

## Data Availability

The datasets generated and/or analyzed during the current study are not publicly available due to a lack of informed consent for data sharing. However, they are available from the corresponding author upon reasonable request.

## List of abbreviations

PA: Physical Activity
FMS: Fundamental Motor Skills
BMI: Body Mass Index
BF: Body Fat
BIA: Bioelectrical Impedance Analysis
LPA: Light Physical Activity
MVPA: Moderate-to-Vigorous Physical Activity
PDMS-2: Peabody Developmental Motor Scales
SS: Stationary Skills
LS: Locomotor Skills
OMS: Object Manipulation Skills
GMQ: Gross Motor Quartile
FM: Fat Mass
FFM: Fat-free Mass
TGMD-2: Test of Gross Motor Development, 2nd Edition
